# Updating targets for natural killer/T-cell lymphoma immunotherapy

**DOI:** 10.20892/j.issn.2095-3941.2020.0400

**Published:** 2021-02-15

**Authors:** Weili Xue, Mingzhi Zhang

**Affiliations:** 1 Department of Oncology, The First Affiliated Hospital of Zhengzhou University, Lymphoma Diagnosis and Treatment Center of Henan, Zhengzhou 450052, China

**Keywords:** Natural killer/T cell lymphoma, immunotherapy, molecular targets

## Abstract

Natural killer/T-cell lymphoma (NKTCL) is a highly invasive subtype of non-Hodgkin lymphoma, typically positive for cytoplasmic CD3, CD56, cytotoxic markers, including granzyme B and TIA1, and Epstein-Barr virus (EBV). The current treatment methods for NKTCL are associated with several drawbacks. For example, chemotherapy can lead to drug resistance, while treatment with radiotherapy alone is inadequate and results in frequent relapses. Moreover, hematopoietic stem cell transplantation exhibits limited efficacy and is not well recognized by domestic and foreign experts. In recent years, immunotherapy has shown good clinical results and has become a hot spot in cancer research. Clinical activity of targeted antibodies, such as daratumumab (anti-CD38 antibody) and brentuximab vedotin (anti-CD30 antibody), have been reported in NKTCL. Additionally, dacetuzumab and Campath-1H have demonstrated promising results. Further encouraging data have been obtained using checkpoint inhibitors. The success of these immunotherapy agents is attributed to high expression levels of programmed death-ligand 1 in NKTCL. Furthermore, anti-CCR4 monoclonal antibodies (mAbs) exert cytotoxic actions on both CCR4+ tumor cells and regulatory T cells. Depletion of these cells and the long half-life of anti-CCR4 mAbs result in enhanced induction of antitumor effector T cells. The role of IL10 in NKTCL has also been investigated. It has been proposed that exploitation of this cytokine might provide potential novel therapeutic strategies. Cellular immunotherapy with engineered cytotoxic T lymphocytes targeted against LMP1 and LMP2 has shown promising results and sustained remission. Cellular immunotherapy may be used either as maintenance therapy following initial induction chemotherapy or in cases of relapsed/refractory disease. The present review outlines the known immunotherapy targets for the treatment of NKTCL.

## Introduction

Natural killer/T-cell lymphoma (NKTCL) is a rare and aggressive disease characterized by a high rate of relapse and poor prognosis^1–3^. Previous studies suggested a male predominance in NKTCL and a median age at diagnosis of approximately 65 years^4–6^. The disease is particularly prevalent in Asia and Latin America^7,8^. The geographic distribution may in part be explained by the incidence of the Epstein-Barr virus (EBV) and the associated aberrant expression of its receptor, namely CD21^9^. NKTCL predominantly occurs in extranodal sites, including the nasal or paranasal areas, and less frequently as a localized nodal lesion. Most tumor cells exhibit a cytotoxic phenotype, primarily characterized by the expression of granzyme B and perforin.

**Figure 1:**
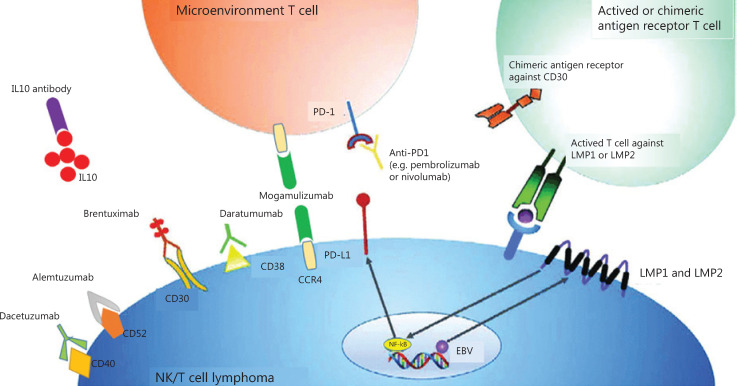
Summary of possible immunotherapy targets in NKTCL and their respective cellular membrane targets. Antibody drugs target cellular membrane proteins, which include brentuximab/CD30, daratumumab/CD38, dacetuzumab/CD40 and alemtuzumab/CD52. Anti-PD1 antibodies, such as pembrolizumab and nivolumab, target microenvironment T-cells that become inactivated and anergy when combined with PD-L1 expressed on tumor cells. Anti-CCR4 antibody mogamulizumab targets not only tumor cells inducing robust ADCC, but Treg cells inhibiting CCL22/CCR4 to reduce lymphoid infiltrates surrounding tumors. LMP1 is a transmembrane protein produced by EBV, activating the NF-κB pathway and lead to cell proliferation and lymphomagenesis. Autologous T cells directed to the LMP2 or LMP1 and LMP2 antigens could induce sustained complete responses without significant toxicity. This was an originally produced image.

The current treatment methods for NKTCL are associated with numerous drawbacks. Early anthracycline-based chemotherapy, such as cyclophosphamide, adriamycin, vincristine, and prednisone, resulted in poor outcomes. This was attributed to the intrinsic resistance of NKTCL cells to anthracycline caused by high expression of P-glycoprotein. Subsequent application of combined modality therapy and concurrent or sequential radiation therapy for the treatment of early stage disease as well as non-anthracycline-based chemotherapy regimens consisting of drugs independent of P-glycoprotein significantly improved the clinical outcomes. Notably, as NK cells lack asparagine synthase activity, neoplasms show high sensitivity toward l-asparaginase^10^. Allogeneic hematopoietic stem cell transplantation (HSCT) has shown potential in patients with stage III/IV of the disease as well as in relapsed patients; however, this approach has limited efficacy. The outcomes of patients with advanced stage disease or those with relapsed/recurrent disease are poor, with overall survival (OS) of just a few months. For disseminated and refractory cases, the 5-year survival rate has been determined as < 10%^11^. Thus, the development of novel effective therapies for this patient population is necessary. In the current review, we evaluate the currently available literature and case reports on immunotherapy options in both frontline and relapsed/refractory NKTCL (**Figure 1**).

## Immunotherapy targets for natural killer/T cell lymphoma

### CD30

The relationship between CD30 expression and the prognosis or clinical characteristics of NKTCL remains unclear. CD30 has been shown to be a predictor of better survival. For example, Kim et al.^12^ reported that the expression of CD30 was significantly and independently associated with longer OS. In their study, 72 patients with NKTCL were treated with non-anthracycline-based chemotherapy. Similarly, Li et al.^13^ suggested that CD30 was an independent prognostic factor for both OS and progression-free survival (PFS); however, it was not related to the treatment response. Moreover, the results of a meta-analysis including 10 retrospective cohort studies with 310 patients were analogous^14^. Nonetheless, in the last 2 years, different outcomes have been reported. For example, Feng et al.^15^ showed that CD30 was frequently expressed in NKTCL and that the expression of this protein was not associated with clinicopathological features or the prognosis. Furthermore, Kawamoto et al.^16^ found that the frequency of CD30 expression was significantly higher in the non-nasal type NKTCL than in the nasal type disease. However, the expression of CD30 was not a prognostic factor for either OS or PFS.

One clinical trial (NCT01309789) showed that a CD30 antibody, namely brentuximab vedotin (BV), resulted in stable remission in patients with CD30-positive peripheral T-cell lymphoma. In addition, CD30-chimeric antigen receptor-transduced T cells were demonstrated as promising new treatment for patients with relapsed or refractory Hodgkin lymphoma^17^. Thus, CD30 should be a useful therapeutic target in the treatment of NKTCL. More recent studies on BV further confirmed promising results regarding its application in NKTCL. For example, a 63-year-old male with CD30-positive NKTCL in the skin was refractory to most chemotherapy regimens. However, after 4 cycles of single agent BV therapy, he showed complete remission^18^. Moreover, a 17-year-old female with NKTCL was initially in complete remission after receiving chemotherapy. The patient then relapsed, and after BV and bendamustine treatment, followed by HSCT, she eventually experienced complete remission^19^. The above outcomes demonstrate that CD30 is a promising immunotherapy target. So far, there have been 2 encouraging clinical trials using the CD30 antibody and CD30-chimeric antigen receptor-engineered T (CART) cells in the treatment of NKTCL (NCT02588651 and NCT03049449, respectively).

### CD38

The expression of CD38 is detected in a majority of patients with NKTCL, which was first demonstrated based on clinical data from 94 patients. Among them, 47 patients exhibited weak expression of CD38, while the other 47 showed strong expression of the glycoprotein. The weak CD38 expression group tended to have a higher incidence of complete remission. Multivariate survival analysis revealed that strong expression of CD38 was an independent adverse prognostic factor for PFS, and correlated with inferior survival outcomes^20^. Hari et al.^21^ classified 10 NKTCL cell lines into CD38^Hi^, CD38^Mid^, and CD38^Low^ groups according to the mRNA and protein levels of CD38 expression. The occurrence of a synergistic cytotoxic effect in CD38^Hi^ cell lines mediated by L-asparaginase treatment in combination with the anti-CD38 antibody, daratumumab, was observed. Daratumumab monotherapy induced sustained remission in NKTCL patients. Clinical trial NCT02927925 showed that daratumumab monotherapy resulted in an encouraging response in patients with relapsed and refractory NKTCL. Overall, the above results showed the potential of single or combined daratumumab treatment for the treatment of relapsed and refractory NKTCL.

In addition to daratumumab, other CD38-specific antibodies are currently in clinical development. These include isatuximab (SAR650984)^22^ and MOR202, whose application has been studied in the treatment of various CD38-positive hematological malignancies. However, the use of these antibodies in NKTCL has not been investigated^23^. Moreover, several CD38-targeting antibodies are in preclinical development, e.g., Ab79 and Ab19 developed by Takeda, the CD3/CD38 bispecific antibody program used by Xencor, and the anti-CD38 antibody drug conjugate (MT-4019) used by Molecular Templates^23^.

In addition to the CD38 antibody, CD38-CART cells have also been recently evaluated. CD38-CART cells appeared to lyse the CD38-positive fractions of CD34-positive hematopoietic progenitor cells, monocytes, natural killer cells, and to a lesser extent, T and B cells. However, they did not inhibit the outgrowth of progenitor cells into various myeloid lineages. These outcomes show the potential of CD38-CART cells as therapeutic tools for CD38-positive malignancies^24^.

Although numerous CD38 antibody and CD38 CART therapies are currently in clinical or preclinical trials^25^, only a few studies have explored their application in NKTCL. Hence, further research into the use of anti-CD38 antibodies or CARTs in this highly aggressive cancer is required.

### CD40

CD40 is a type I transmembrane protein, which belongs to the tumor necrosis factor receptor (TNFR) family. CD40 is expressed on B cells, monocytes, dendritic cells, endothelial cells, and epithelial cells, and plays a critical role in the regulation of immune responses^26^. It has been reported that EBV infections induced CD40 expression in T cells^27^. CD40 is expressed on the surface of EBV-infected T and NK cell lines, including SNT8, SNT13, SNT15, SNT16, SNT20, SNK1, SNK6, and SNK10^28^. In cutaneous T-cell lymphoma, CD40/CD40 ligand interactions have been shown to exhibit a paracrine loop, which is crucial for prevention of apoptosis or positive regulation of growth. It is also key in the process of homing neoplastic cells in the skin^29^. In mouse T-cell lymphoma, the anti-CD40 antibody has been found to induce antitumor and anti-metastatic effects^30^. Notably, CD40/CD40L signaling plays an important role in the pathogenesis of NKTCL associated with EBV^28^.

The CD40/CD40L interaction leads to the activation of dendritic cells (DCs), which results in the secretion of the proinflammatory cytokine IL-12, supports CD4+ T cell helper responses, and CD8+ cytotoxic T cell priming^31–33^. Moreover, CD19-CART cells constitutively expressing CD40L have been shown to exhibit superior antitumor efficacy, while approved antigen-presenting cells enhanced the recruitment of immune effectors and mobilized endogenous tumor-recognizing T cells in B-cell malignancies^34,35^. Treatment with dacetuzumab (SGN-40), a humanized IgG1 mAb targeting CD40, did not achieve improved complete remission when combined with rituximab and ifosfamide-carboplatin-etoposide (R-ICE) in relapsed, diffuse, large B-cell lymphoma (DLBCL) in a phase 2 study (NCT00529503). Overall, the application of the anti-CD40 antibody or CART cells targeting CD40 in the treatment of NKTCL should be explored further.

### CD52

The anti-CD52 antibody, alemtuzumab, has been explored as a novel targeted therapy for T-cell malignancies. However, the expression of CD52 in NKTCL has not been extensively studied. Walewski et al.^36^ suggested that only some of the tumor cells expressed the CD52 antigen in 6 NKTCL samples. In addition, Chang et al.^37^ reported that 8/17 (47%) NKTCL patients expressed CD52. Similarly, Jiang et al.^38^ examined 4 NKTCL cases and found that only one was positive for CD52. Besides NKTCL, a comprehensive study on the expression of CD52 in 74 patients diagnosed with mature NKTCL was also conducted. It was concluded that CD30 expression was frequently associated with the absence of CD52 expression. However, to effectively use the CD52 and CD30 antibodies in the treatment of NKTCL, further studies are needed to support the above studies. Lefrançois et al.^39^ showed that CD52 was associated with robust disease progression and decreased survival biomarkers in cutaneous T-cell lymphoma patients.

Notably, clinical trials involving the application of the anti-CD52 antibody, alemtuzumab (NCT00069238, NCT00040846, and NCT00118352), in NKTCL are currently being conducted. 

### PD1/PD-L1

Inhibiting the programmed cell death ligand 1 (PD-L1) pathway has emerged as a promising strategy for cancer therapy. The expression of PD-L1 in NKTCL ranges from 39% to 100%^40–44^. In addition, the expression of PD-L1 has been shown to be significantly higher in EBV-positive than in EBV-negative non-Hodgkin lymphoma (NHL)^45^. However, it is unclear whether the expression of PD-L1 affects the OS or other clinical parameters. Jo et al.^41^ reported that there was no significant difference in OS between PD-L1-positive and PD-L1-negative NKTCL patients. Conversely, other studies revealed that patients with PD-L1-positive NKTCL exhibited a better 5-year OS and longer PFS^40,41,43^. Furthermore, NKTCL patients with high PD-L1 expression showed an enhanced response compared to those with low expression of the protein. In terms of clinical features, the majority of studies agree that the expression of PD-L1 affects certain clinical parameters, such as international prognostic index (IPI) and lactate dehydrogenase (LDH)^41,44^. However, Kim et al.^40^ reported that PD-L1 expression did not influence the clinicopathological features. Thus, further studies involving larger sample sizes are required to clarify the contrasting findings.

Pembrolizumab is a PD-1 monoclonal antibody first approved in 2014 by the US Food and Drug Administration for the treatment of metastatic melanoma. Although pembrolizumab has been used to treat various subtypes of NHL, real-world data on the efficacy of pembrolizumab in NKTCL patients are limited. Although 3 studies published some data about clinical trials, the sample sizes were small. For example, 1 of the studies included just 7 male patients diagnosed with NKTCL, who did not respond to chemotherapy and allogeneic hematopoietic stem-cell transplantation (HSCT). The patients were treated with pembrolizumab, and complete remission was observed in 5 patients. In addition, partial response was noted in 2 patients^46^. Another study was a case report, in which a 37-year-old female with NKTCL did not respond to continuous chemotherapy followed by radiotherapy. The patient was treated with 4 cycles of pembrolizumab, which resulted in complete remission^47^. The third trial involved 7 patients with a diagnosis of NKTCL. Similar to the above studies, the patients previously did not respond to chemotherapy. During the trial, they were treated with 4 cycles of pembrolizumab. Complete remission was observed in 2 patients, while partial remission was noted in 2 patients^48^. Overall, inhibition of PD-1 with pembrolizumab is a favorable strategy for the treatment of refractory or relapsed NKTCL^49^. However, its efficacy in EBV-negative NHL with low or absent PD-L1 expression remains unclear. It is noteworthy that pembrolizumab could be a potential treatment option for relapsed or refractory NHL^45^.

The results of the above studies indicate that pembrolizumab could be a useful alternative treatment for relapsed or refractory NKTCL. Nevertheless, the development of cutaneous CD56-positive T-cell lymphoma has been reported during pembrolizumab treatment for metastatic melanoma^50^. Although the occurrence of lymphoma in this case cannot be fully attributed to the treatment with pembrolizumab, this reason cannot be excluded. Thus, any anti-PD1 treatment, particularly pembrolizumab, should be carefully considered prior to treatment, and, implementation of further clinical trials with larger sample sizes is essential. In addition, a PD-L1 mutation and a diverse baseline T cell receptor (TCR) repertoire have been proved a potential biomarkers to better select patients with NKTCL for anti-PD-1 therapy^51,52^. Before receiving immune checkpoint inhibitor therapy, NKTCL patients could be analyzed for PD-L1 mutations and a TCR sequence to avoid economic excess and minimize adverse events. At present, multiple clinical trials aim to assess the efficacy of anti-PD1 therapies in NKTCL, including early stage (NCT03728972) and relapsed or refractory NKTCL (NCT03021057 and NCT03107962, and NCT03586024).

In most cases, no immune deficiency is observed in patients with NKTCL, strongly suggesting that tumor cells develop strategies to escape the strong immune response established against EBV antigen-expressing cells. Similar to other EBV-associated neoplasias, such as nasopharyngeal lesions, gastric carcinoma, and Hodgkin lymphoma, the expression of PD-L1 by NK/T lymphoma cells is likely to be involved in this resistance. The combination of frequent PD-L1 expression and the presence of foreign antigen expression in tumor cells makes the use of checkpoint inhibitors an attractive treatment alternative^53^. It is speculated that PD-L1 could be upregulated by EBV-driven LMP1 through the NF-κB pathway^54^ and signal transducer and activator of transcription 3 (STAT3) activation, providing the rationale to combine STAT3 inhibitors or anti-EBV agents with immune checkpoint inhibitors^55^. The combination of anti-PD1 agents with BV could also further potentiate antitumor activity.

### CCR4

CC chemokine receptor 4 (CCR4) plays an important role in regulating immune balance and is known to be highly expressed in Treg cells, particularly CD45RA^−^FOXP3^hi^CD4^+^ Treg cells [designated as effector Treg (eTreg) cells]. However, CD45RA^+^FOXP3^lo^CD4^+^ naive Treg cells, CD8^+^ T cells, NK cells, CD14-positive monocytes/macrophages, dendritic cells, and B cells exhibit low CCR4 expressions. Moreover, some T cell lymphoma cells express CCR4^56,57^. CCR4 is a receptor for the CCL22 and CCL17 chemokines, which are mainly produced by DCs and macrophages. The CCR4-CCL22/CCL17 interaction plays a major role in Treg migration and metastasis of CCR4-positive lymphoma cells. Treg cells recruited through CCL22/CCR4 are selectively activated in lymphoid infiltrates surrounding primary breast tumors and lead to an adverse clinical outcome^58^. Additionally, Th2 and a small population of Th17 cells also express CCR4; however, recruitment of these cells to tumor sites is associated with a poor prognosis^59^.

It has been shown that CCR4 is expressed in several NKTCL cell lines (e.g., KAI3, NKL, HANK1, SNK10, SNK6, SNT8, SNK1, SNT1, and SNT6)^60–63^. Kumai et al.^60^ confirmed the expression of CCR4 in 5 patients. It was found that in 2 patients, > 10% of the CD56-positive tumor cells were CCR4-positive. Moreover, Berahovich et al.^61^ analyzed peripheral blood mononuclear cells from 11 donors. It was determined that CCR4 was expressed in 3.6% of CD56 bright NK cells and 6.8% of CD56 dim NK cells. Notably, the expression of CCR4 was downregulated upon treatment with vorinostat, a pan-histone deacetylase inhibitor^62^.

In 2018, the US Food and Drug Administration approved the mogamulizumab-CCR4 antibody for adult patients with relapsed or refractory mycosis fungoides or Sézary syndrome after at least one prior systemic therapy. Mogamulizumab induced robust antibody-dependent cellular cytotoxicity against NKTCL cell lines^60^. Tumor growth was significantly suppressed in the mogamulizumab-treated group in 1 NK-cell lymphoma mouse model^64^. Furthermore, mogamulizumab showed notable antitumor activity in adult T-cell leukemia/lymphoma in a phase II study (NCT00920790)^65^. The above findings suggest that CCR4 might be a useful target for the treatment of NKTCL.

Anti-CCR4 mAbs exert cytotoxic action on both CCR4-positive tumor cells and Tregs. Additionally, Tregs and tumor cells as well as the long half-life of anti-CCR4 mAbs favor induction of antitumor effector T cells. Hence, CCR4 represents an ideal target to block both Tregs and CCR4-positive lymphoma cells. Further investigation is necessary to validate the hypotheses and to elucidate the mechanisms involved in the activity of mogamulizumab. Moreover, enhancement of antitumor responses in non-CCR4 tumors by anti-CCR4 mAbs should be studied.

### IL-10

Interleukin 10 (IL-10) is a cytokine expressed by numerous cell types, including innate and adaptive immune cells^66^. Its expression is precisely controlled in cells by transcriptional and post-transcriptional regulations. It has been shown that IL-6 or IL-27 in conjunction with anti-CD3 and anti-CD28 Abs induces the expression of IL-10 in both CD4-positive and CD8-positive T cells. In addition, IL-10 expression is dependent on STAT1 in IL-27-stimulated T cells^67^. Intriguingly, STAT3 activation enhances IL-10 expression^68^. Epigenetic factors, such as histone modification, have also been found to affect the expression of this cytokine^69,70^. Studies have shown that the expression of IL-10 could also be regulated at the post-transcriptional level. The IL-10 gene contains destabilizing AUUA repeats within its 3′-UTR. These motifs are thought to be recognized by the RNA binding protein tristetraprolin, which facilitates the degradation of mRNA. It has been proposed that some cells might constitutively transcribe the IL-10 gene and regulate its expression at the post-transcriptional level to shorten the signal response time^70^.

The complex regulation process results in pleiotropic biological activity of IL-10, which plays a dynamic role in the tumor microenvironment. For example, IL-10 produced by B-cell lymphoma cells serves as an autocrine growth factor^71^. Inhibition of IL-10 signaling at the time of immunization promotes the generation of robust vaccine-induced CD8 T responses, which are able to inhibit tumor growth. It has been reported that inhibition of CTLA-4 induces anti-tumor activity through decreasing IL-10 secretion^72^. IL-10 signaling in the tumor infiltrating CD8 T cells is also able to inhibit tumor growth^73^. Thus, the role of IL-10 signaling tumor immunotherapy remains somewhat ambiguous. It is known that stimulation and inhibition of IL-10 signaling can prevent tumor growth. Hence, careful selection of target cells, inhibition of IL-10 signaling at the time of immunization, and activation of IL-10 signaling at the tumor site could lead to a better cancer therapy outcome.

Only a few studies on the significance of IL-10 in NKTCL have been reported. Boulland et al.^74^ found that 3 out of 7 NKTCL patients participating in the study exhibited a large number of IL-10 expressing cells. However, Shen et al.^75^ reported that IL-10 mRNAs were present in all tested tumors, and that IL-10 protein expression was detected in the majority of tumor cells isolated from 13 patients with NKTCL. Furthermore, another study showed that among 98 newly diagnosed NKTCL patients receiving asparaginase-based chemotherapy, those with high levels of IL-10 at diagnosis tended to have more adverse clinical features. In contrast, subjects with low IL-10 expression had better PFS and OS. Multivariate analysis showed that a high baseline serum IL-10 level was an adverse factor for PFS and OS^76^. Similarly, Cai et al.^77^ reported that IL-10 expression was high in the majority of 59 studied NKTCL cases. The serum concentration of IL-10 was higher in patients with fever and advanced stage of the disease as well as in those with NF-kB activation involving an alternative pathway (P52). Moreover, the levels of IL-10 dramatically increased in 10 patients with hemophagocytic syndrome. In contrast to the previous study, multivariate analysis did not show that IL-10 could be used as a predictor of PFS and OS as an independent prognostic factor.

IL-10 is a novel and powerful prognosis predictor for NKTCL, which implies an important role of this cytokine in the pathogenesis of the disease, providing new insights into potential therapeutic strategies.

### EBV-related antigens

EBV plays an important role in lymphomagenesis of NKTCL. The latent genes in EBV-infected T and NK cells are restricted in Epstein-Barr virus-encoded small RNAs, Epstein-Barr virus nuclear antigen 1 (EBNA1), latent membrane protein (LMP1), and LMP2^78–80^. The correlation between EBV and NKTCL has been previously studied^81^. It has been found that the virus was present in nearly all patients, irrespective of their ethnicities^81–83^. It addition, it was determined that all tumor cells within a lesion contained EBV genomes, and that the virus was clonal^81,84,85^. Importantly, EBV-encoded transcripts and proteins have also been detected^85,86^.

Kanemitsu et al.^87^ suggested that LMP1 and LMP2A were present in 22 and 12 out of 30 NKTCL cases, respectively. Notably, the LMP1-positive cases exhibited a better 2.5-year OS. In their study, Schwartz et al.^88^ established the presence of EBV in 73 out of 84 NKTCL cases. Furthermore, Zhao et al.^89^ reported that the total LMP1 expression was 83.58% in 67 NKTCL cases. No statistically significant difference was observed between the expressions of LMP1 and the disease prognosis. Ian et al.^90^ confirmed that a low level of p27 protein expression could be partially attributed to latent EBV infection in NKTCL. They also showed that EBNA1 might be a suitable target for the treatment of EBV-associated NKTCL.

The expression of EBV proteins in the tumor provides targets for adoptive immunotherapy with antigen-specific cytotoxic T cells (CTL). Viral antigen targeting through autologous T cell activation has been applied in the treatment of viral reactivation following bone marrow transplants and EBV-associated post-transplant lymphoproliferative disorder^91–94^.

Several clinical trials (NCT00062868 and NCT01956084) evaluated the efficacy of donor-derived LMP-specific T cells (LMP-Ts) in 26 patients with EBV-associated NK/T-cell or B-cell lymphomas. The 2-year OS was determined to be 68%^95^. Another study showed that in NKTCL patients, autologous T cells directed to the LMP2 or LMP1 and LMP2 antigens could induce sustained complete responses without significant toxicity. After induction therapy, 10 NKTCL patients were treated with EBV LMP-1 and LMP-2a-specific CTLs (LMP1/2a CTLs) stimulated with LMP1/2a RNA-transferred dendritic cells (NCT00671164). In this case, the 4-year OS and PFS were established at 100% and 90%, respectively^96^. These studies indicated that autologous T cells directed to the LMP antigen could induce sustained complete remission (CR) in NKTCL patients, demonstrating that EBV proteins could act as effective targets for NKTCL immunotherapy.

### CD137

EBV also promotes the expression of CD137, which after stimulation by its ligands inhibits the apoptosis of EBV-positive T/NK cells^97^. *In vitro* IL-2 treatment was shown to enhance the expression of CD137 in EBV-positive cells. Yoshimori et al.^97^ implied that LMP1 induced the synthesis of IL-2, stimulating the expression of CD137. In addition, it was found that LMP1 stimulated the expression of CD137 through AP-1 and NF-κB^98^.

Several studies imply that under normal conditions, CD137 limits EBV in causing pathology. Alosaimi et al.^99^ identified a CD137 mutation in 2 unrelated patients with recurrent infections, involving persistent EBV viremia and EBV induced lymphoproliferation. The mutation abolished surface expression of CD137 on activated T cells, resulting in a diminished proliferation, IFN-γ secretion, perforin expression, and a reduced cytotoxic activity of CD8+ T cells upon stimulation with allogeneic and human leukocyte antigen (HLA)-matched EBV-transformed B cells. Somekh et al.^100^ reported that a mutation in CD137 was the cause of the reduced T cell proliferation capacity. Rodriguez et al.^101^ concluded loss of CD137 expression impaired T cell expansion.

On the basis of these results, CD137 agonists should be studied for the treatment of EBV-associated malignancies such, as NKTCL^102^. Hence, CD137 could act as another potential immunotherapy target for NKTCL.

## Conclusions

Better understanding of the lymphoma microenvironment has resulted in the development of numerous immune therapy strategies. Such therapies enable clinicians to perform precision medicine and significantly improve the prognoses of patients. Nevertheless, many aspects, such as treatment scheduling, dosage, and combinations with other agents, remain unclear. Moreover, the identification of potential biomarkers, which could be used to predict the clinical responses and toxicities of the available targeted therapies, is challenging. However, immunotherapy combined with other mechanism-based targeted therapies is a promising strategy to eventually make NKTCL a curable disease.
